# Magnetic resonance radiomics signatures for predicting poorly differentiated hepatocellular carcinoma: A SQUIRE-compliant study

**DOI:** 10.1097/MD.0000000000025838

**Published:** 2021-05-14

**Authors:** Xiaozhen Yang, Chunwang Yuan, Yinghua Zhang, Zhenchang Wang

**Affiliations:** a Department of Center of Interventional Oncology and Liver Diseases, Beijing Youan Hospital; b Department of Radiology, Beijing Friendship Hospital, Capital Medical University, Beijing, China.

**Keywords:** hepatocellular carcinoma, low differentiation, magnetic resonance imaging, radiomic signature

## Abstract

Radiomics contributes to the extraction of undetectable features with the naked eye from high-throughput quantitative images. In this study, 2 predictive models were constructed, which allowed recognition of poorly differentiated hepatocellular carcinoma (HCC). In addition, the effectiveness of the as-constructed signature was investigated in HCC patients.

A retrospective study involving 188 patients (age, 29–85 years) enrolled from November 2010 to April 2018 was carried out. All patients were divided randomly into 2 cohorts, namely, the training cohort (n = 141) and the validation cohort (n = 47). The MRI images (DICOM) were collected from PACS before ablation; in addition, the radiomics features were extracted from the 3D tumor area on T1-weighted imaging (T1WI) scans, T2-weighted imaging (T2WI) scans, arterial images, portal images and delayed phase images. In total, 200 radiomics features were extracted. *t* test and Mann–Whitney *U* test were performed to exclude some radiomics signatures. Afterwards, a radiomics signature model was built through LASSO regression by RStudio Software. We constructed 2 support vector machine (SVM)-based models: 1 with a radiomics signature only (model 1) and 1 that integrated clinical and radiomics signatures (model 2). Then, the diagnostic performance of the radiomics signature was evaluated through receiver operating characteristic (ROC) analysis.

The classification accuracy in the training and validation cohorts was 80.9% and 72.3%, respectively, for model 1. In the training cohort, the area under the ROC curve (AUC) was 0.623, while it was 0.576 in the validation cohort. The classification accuracy in the training and validation cohorts were 79.4% and 74.5%, respectively, for model 2. In the training cohort, the AUC was 0.721, while it was 0.681 in the validation cohort.

The MRI-based radiomics signature and clinical model can distinguish HCC patients that belong in a low differentiation group from other patients, which helps in the performance of personal medical protocols.

## 1 Introduction

Liver cancer ranks fifth in terms of its morbidity, and it is also the second leading cause of cancer-related death in the world. Hepatocellular carcinoma (HCC) represents approximately 90% of primary liver cancers, rendering it a major global health issue.^[1]^ Hepatitis represents the leading etiology of primary liver cancer, and most hepatitis patients are found in China. Recurrence is a key point in the treatment of HCC patients; recurrence not only adds to the mental pressure and financial burdens of patients but also results in distrust of medical tactics. The pathological type of HCC is important for predicting overall survival (OS). Low differentiation is 1 pathological type of HCC that always recurs and metastasizes quickly. Low differentiation makes cancer recovery difficult, which reduces the OS of patients. Therefore, discriminating patients with low differentiation HCC can prompt us to pay more attention to follow-up and to implement supplementary proposals to prolong patient OS. Biopsy or hepatectomy is the only way to determine the pathological type, which is further confirmed by pathological examination. A decompensated cirrhosis patient is not fit for this invasive operation; the procedure for this operation can be improved and made more suitable for these patients by this noninvasive method used in our study. This can also reduce the number of metastasis cases related to biopsy processes. A prediction model for lowly differentiated HCC was constructed in this study, which was used to distinguish the poor prognosis group from other groups, thus contributing to the formulation of feasible individual medical plans.

Radiomics contributes to the extraction of the high-dimensional and high-throughput quantitative features from imaging, which are not visible. Typically, radiomics can obtain imaging information that cannot be detected by the naked eye and is beyond our perception. At present, increasing efforts have been made concerning radiomics since the original investigation in 2012.^[2]^ In addition, “radiomics” has become more prevalent since it was defined, which can be ascribed to its noninvasiveness, variable modalities, quantitative image features, and dimension. Radiomics techniques (including CT, MRI, PET-CT, and US) have been applied in predicting recurrence, treatment outcomes, and survival, as well as in differentiating similar appearance imaging features.^[3]^ Initially, lung cancer, colon cancer, glioma, and breast cancer were investigated using radiomics techniques; to date, radiomics techniques have been applied to multiple pertinent fields, such as bone tumors and liver tumors.^[4,5]^ The studied radiomics features for HCC include early recurrence, prognosis, survival, and microvascular invasion.^[6–8]^

Notably, considerable efforts have been made in precision medicine, which makes personalized medicine feasible. Several methods can be used to construct a radiomics model. To date, no consensus has been reached on a radiomics strategy; however, radiomics features are of vital importance for oncology.

To the best of our knowledge, no favorable noninvasive approach is available for patient stratification according to pathological differentiation. A radiomics signature can quantify a signature and highlight pathological information, while the images can offer the whole-lesion features. To date, few studies are available regarding MRI radiomics features to predict the poorly differentiated pathological type of HCC. In our study, 1 radiomics feature was extracted from all the radiomics features to construct a radiomics model. Clinical indexes and the radiomics signature were combined to establish another model. In addition, the pathological stratification effect of the radiomics model on HCC was also investigated with the aim of supplementing the MRI images.

## 2 Materials and methods

### 2.1 Patients

The present study was conducted in accordance with the Declaration of Helsinki and was approved by the Ethics Committee of Beijing Youan Hospital (2018010).

A total of 188 HCC patients referred to our hospital from November 2010 to April 2019 were enrolled in this study and randomly divided into a training cohort (n = 141; including 114 males and 27 females, with an age of 57.86 ± 10.934 years) and a validation cohort (n = 47; including 36 males and 11 females, with an age of 58.34 ± 11.316 years). The patient inclusion criteria were as follows:

1. patients with HCC revealed by biopsy results,
2. patients treated with ablation (either imaging-guided or under laparoscopy), and
3. patients who underwent enhanced MRI scanning before treatment.

The patient exclusion criteria were as follows: patients with no available biopsy results or who did not undergo enhanced MRI imaging. The following patient information was recorded: alanine transaminase level, aspartate aminotransferase level, platelet level, and alpha fetoprotein level, load of virus, diameter of tumor, number of tumors, and OS. MRI was performed from PACS before treatment. The study was completed in March 2019.

### 2.2 Lesion segmentation

The images were derived from the PACS of Beijing Youan Hospital and were then segmented using ITK-SNAP software. In addition, ITK-SNAP was used to manually delineate lesions slice by slice by 1 experienced radiologist blinded to the pathological results and clinical data. If there were 3 lesions, the largest lesion was delineated. There were 5 phases to be segmented, including T1-weighted imaging (T1WI), T2-weighted imaging (T2WI), arterial, vein-portal and delay phases. The other information recorded was from biological reports and laboratory test results. The patients were divided into 2 groups: the training (n = 141) and validation (n = 47) groups.

### 2.3 Feature extraction

Radiomics features were extracted using LIFEx software, including first-order and second-order features. A total of 200 candidate features were generated from each patient, with 38 features from images at each phase. Table 1 displays the radiomics features included in the analysis. LIFEx software was employed to develop the analysis algorithms for all feature extraction methods.

**Table 1:** Radiomics features included in our analysis.

Types		Features
First order features	Shape (n = 3)	SHAPE _Sphericity
		SHAPE _Compacity
		SHAPE _Volume (mL)
	Histogram (n = 5)	HISTO _Skewness
		HISTO _Kurtosis
		HISTO _Entropy _log10
		HISTO _Entropy _log2
		HISTO _Energy
Second order features	GLCM (n = 7)	GLCM _Homogeneity
		GLCM _Energy
		GLCM _Contrast
		GLCM _Correlation
		GLCM _Entropy _log10
		GLCM _Entropy _log2
		GLCM _Dissimilarity
	NGLDM (n = 3)	NGLDM _Coarseness
		NGLDM _Contrast
		NGLDM _Busyness
	GLRLM (n = 11)	GLRLM _SRE, GLRLM _LRE
		GLRLM _LGRE, GLRLM _HGRE
		GLRLM _SRLGE, GLRLM _SRHGE
		GLRLM _LRLGE, GLRLM _LRHGE
		GLRLM _GLNUr, GLRLM _RLNU
		GLRLM _RP
	GLZLM (n = 11)	GLZLM _SZE, GLZLM _LZE
		GLZLM _LGZE, GLZLM _HGZE
		GLZLM _SZLGE, GLZLM _SZHGE
		GLZLM _LZLGE, GLZLM _LZHGE
		GLZLM _GLNUz, GLZLM _ZLNU
		GLZLM _ZP

### 2.4 Construction of the radiomics signature model

LASSO was performed to reduce redundancy in the evaluation of the potential relationships between radiomics features and the low differentiation in both the training and validation cohorts. Radiomics features were retained if *P* < .05 (2-sided). One radiomics feature was ultimately selected. To reduce the variables, LASSO was used to select the significant variables. Afterwards, the selected features and clinical indexes were used to establish the models, and the low differentiation possibility of each patient was determined according to the radiomics signature. The receiver operating characteristic (ROC) curve was used to quantify discrimination performance.

### 2.5 Statistical analysis

Data were analyzed using SPSS statistics 19.0 (IBM, Armonk, New York) and R Studio software. In addition, the Shapiro–Wilk approach was utilized to test the distribution normality of continuous variables. Normally distributed data were recorded as the median ± standard deviation and analyzed by Student *t* test. Data not conforming to a normal distribution were recorded as the median (range) and analyzed by the Mann–Whitney *U* test. Additionally, categorical data were recorded as frequencies and analyzed by the Chi-Squared test. A difference of *P* < .05 (2-sided) was deemed statistically significant.

## 3 Results

### 3.1 Clinical characteristics

The clinical characteristics of the training and validation cohorts are presented in Table 2. There was no significant difference between the 2 groups according to the Chi-Squared test (*P* = .475), and the low differentiation rates in those 2 groups were 20.1% (training group, 29/141) and 25.5% (validation group, 12/47).

**Table 2:** The clinical information of patients.

	Training cohort (n = 141)	Validation cohort (n = 47)	*P* value
Age (yr)	57.86 ± 10.934	58.34 ± 11.316	.809
Sex			.529
Male	114 (80.9)	36 (76.6)	
Female	27 (19.1)	11 (23.4)	
AFP (ng/mL)	16.515 (25094.096)	9.02 (5448.99)	.245
ALT (U/L)	34 (403.9)	33.7 (119.8)	.530
AST (U/L)	35.7 (293.95)	35.95 (84.7)	.294
PLT(E+9/L)	110 (262)	119 (200)	.406

### 3.2 Construction and validation of the radiomics signature

Patients were randomly divided into 2 groups (training (n = 141) and validation (n = 47). The model prediction ability was validated through the following method. First, missing data were replaced by the average value. Second, significant radiomics signatures were analyzed using *t* tests and MWU tests. Third, 10-fold cross validation was used by LASSO to obtain the radiomics signature, which was used to construct the prediction model. Specifically, 1 radiomics feature, GLZLM_LZHGE, was used to construct the model.

**Figure 1:**
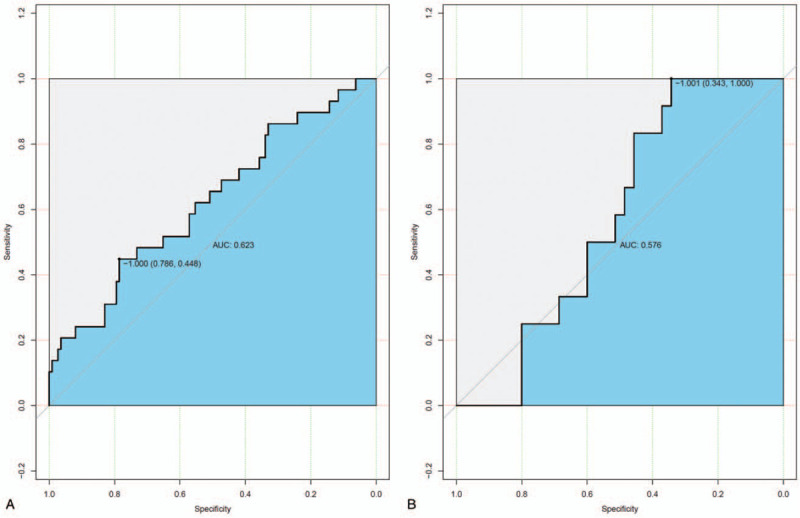
The model 1 ROC curve for the training cohort (A) and the validation cohort (B).

**Figure 2:**
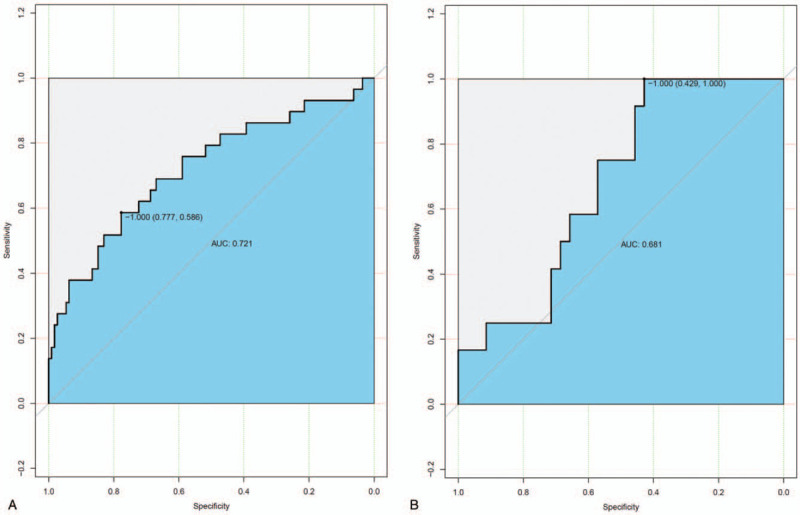
The model 2 ROC curve for the training cohort (A) and the validation cohort (B).

There were 2 models: model 1 was a radiomics signature only, and model 2 was an integration of the clinical index and radiomics signature. Significant differences in radiomics signatures were observed between patients in the low differentiation and nonlow differentiation groups in both cohorts. For model 1, the classification accuracies were 80.9% and 72.3% in the training and validation cohorts, respectively. In the training cohort, the area under the ROC curve (AUC) was 0.623 (95% CI, 0.5052–0.7417, Lasso regression), while it was 0.576 (95% CI, 0.4142–0.7382, Lasso regression) in the validation cohort (Fig. 1A and B). For model 2, the classification accuracies were 79.4% and 74.5% in the training and validation cohorts, respectively. In the training cohort, the AUC was 0.721 (95% CI, 0.6069–0.8353, Lasso regression), while it was 0.681 (95% CI, 0.5215–0.8404, Lasso regression) in the validation cohort (Fig. 2A and B). The AUC is a threshold-independent metric because it evaluates the performance of a model at all possible threshold values.^[9]^ It is the standard method to assess prediction accuracy because of its threshold independence and the ease of interpreting its results.^[10,11]^ The AUC value in the ROC curve of <0.5 suggested no predictability, and an AUC value between 0.51 and 0.7 indicated low accuracy. An AUC value between 0.71 and 0.9 suggested moderate accuracy, and an AUC value of 0.9 indicated high accuracy; the closer to 1, the better the predictability.^[11]^ According to the AUC values, the prediction accuracy in the training group was relatively low but was statistically significant, while that in the validation group was moderate.

## 4 Discussion

In our study, a radiomics signature model was constructed to stratify patients according to their pathological results. Typically, low differentiation signifies progressive biological behaviors, along with faster lesion proliferation, earlier vascular invasion and easy metastasis. All the above mentioned features result in shorter OS times than the features inherent to other pathological types. During an ablation procedure, a security margin should be guaranteed for patients, and rigorous follow-up is needed. When a new lesion is detected, ablation should be applied in the absence of any contraindications. In addition, some adjuvant therapy can be considered.

In our study, the individual radiomics features were GLZLM_LZHGE at the arterial phase. The gray-level zone length matrix (GLZLM) provides information on the size of homogeneous zones for each gray level in 3 dimensions (or 2D). GLZLM_LZHGE was indicative of the distribution of long homogeneous zones with high gray levels. Individual radiomics features were not entirely the same due to the heterogeneities in disease and modality, and tumor heterogeneity was expressed as the distribution pattern of voxels. In addition, tumor biological behaviors were dependent on heterogeneity. Additionally, vascular proliferation, tumor cell necrosis, calcification, and microvascular invasion were related to the differentiation degree of HCC. In addition, the outcome of model 2 was improved compared to that of model 1. Integrating the clinical index and radiomics signature can help discriminate HCC with low differentiation.

The target imaging sequence in this study was different from the sequences reported in other studies. For instance, some studies have used ADC maps and some have focused on T1WI, T2WI, and diffusion-weighted imaging sequences, while other authors may have only focused on T1 post enhanced images. For example, Yuming Jiang proved that a radiomics nomogram predicted survival for gastric cancer, and the adopted signatures were Hist_Var, Hist_Entropy, and LGRE_GLRLM.^[12–17]^ Each imaging modality has its own priority based on different target organs. Specifically, dynamic contrast-enhanced MRI is good for breast cancer, while PET/CT is superior when studying bone tumors. Lung cancer is mainly evaluated by dynamic contrast-enhanced CT, and MRI can offer much more detailed information. Therefore, the imaging modality should be selected based on the subject of investigation. In this study, enhanced MRI features were selected as much as possible to analyze and extract radiomics features. However, some lesion margins were not clear in the diffusion-weighted imaging sequences; as a result, they were not included in our study. In addition, the arterial and delayed phase sequences were quite important for the diagnosis of HCC. Lesion proliferation was reflected by the abovementioned phases.

Our study included many more radiomics features than some studies, suggesting the lower possibility of omitting any key radiomics signature in this study.^[18]^ Some studies only included textual analysis features, and the region of interest was in 3D. Compared with other studies, we delineated every slice of the whole tumor, which added much more information and enhanced the reliability of the results.^[19]^

Additionally, the tumor area was selected as the interesting area, which was commonly used but different from 1 study. In that study, the author analyzed the areas of the tumor and peritumor. We can include peritumor segmentation in the future, which may contribute to new achievements. One study proved that peritumor segmentation better predicted tumor recurrence; therefore, peritumor segmentation might be included in the next step of the study.^[20]^

Multiple modalities and genomics combined studies are needed.^[21–26]^ Each modality has its own advantages. PET/CT can show the distribution of body tracer activities. Ultrasonograms in ultrasound offer heterogeneous and homogeneous information. MRI signal helps to discern different substances. Typically, dynamic contrast-enhanced CT is a useful imaging modality to assess chemotherapy responses due to its high sensitivity to angiogenesis. Moreover, combining different modalities and genomics together can provide information about integral lesion features.

Furthermore, there are several ways to improve prediction accuracy. First, some other indexes, such as molecular markers, could be included in the nomograms. H X Yang et al constructed 8 support vector machine (SVM)-based nomograms and found that SVM-based models integrating clinicopathological features and molecular markers showed higher prediction accuracy than other models.^[27]^ Second, the features extracted from the fusion image could improve prediction performance. Vallieres et al found that the combination of features extracted from PDG-PET and MRI scans had the best performance.^[28]^ In addition, the identification of optimal machine learning methods for radiomic markers could also predict performance, which is a crucial step for providing a noninvasive way of quantifying and monitoring tumor phenotypic characteristics in clinical practice.^[29]^ Finally, there are other methods, such as multicenter validation with a larger sample size, categorizing patients according to tumor size or imaging trials, and analyzing outliers to increase the accuracy; these methods need further validation.

Several limitations should be noted in this study. First, it was a retrospective study with a small sample size. Second, the study cohorts came from our institution alone. Therefore, prospective studies with more samples collected from multiple centers will be needed in the future. In summary, more efforts are warranted in this field.

## 5 Conclusions

In this study, individual radiomics features related to poorly differentiated HCC were identified, which helped to formulate a personal medical protocol for patients with poor prognosis.

## Author contributions

**Conceptualization:** Zhenchang Wang.

**Data curation:** Yinghua Zhang.

**Formal analysis:** Xiaozhen Yang, Yinghua Zhang.

**Funding acquisition:** Zhenchang Wang.

**Investigation:** Chunwang Yuan.

**Methodology:** Xiaozhen Yang, Zhenchang Wang.

**Project administration:** Yinghua Zhang, Zhenchang Wang.

**Resources:** Chunwang Yuan.

**Software:** Xiaozhen Yang, Chunwang Yuan.

**Supervision:** Yinghua Zhang.

**Visualization:** Chunwang Yuan.

**Writing – original draft:** Xiaozhen Yang.

**Writing – review & editing:** Zhenchang Wang.

## References

[1] Galle PR Forner A Llovet JM. EASL clinical practice guidelines: management of hepatocellular carcinoma. J Hepatol 2018;69:182–236. 29628281 10.1016/j.jhep.2018.03.019

[2] Peng J Zhang J Zhang Q. A radiomics nomogram for preoperative prediction of microvascular invasion risk in hepatitis B virus-related hepatocellular carcinoma. Diagn Interv Radiol 2018;24:121–7. 29770763 10.5152/dir.2018.17467 PMC5951199

[3] Hui T Chuah T Low H. Predicting early recurrence of hepatocellular carcinoma with texture analysis of preoperative MRI: a radiomics study. Clin Radiol 2018;73: 1506.e11-1506.e16. 10.1016/j.crad.2018.07.109 30213434

[4] Yuan C Wang Z Gu D. Prediction early recurrence of hepatocellular carcinoma eligible for curative ablation using a Radiomics nomogram. Cancer Imaging 2019;19:21. 31027510 10.1186/s40644-019-0207-7 PMC6485136

[5] Shan Q-y Hu H-t Feng S-t. CT-based peritumoral radiomics signatures to predict early recurrence in hepatocellular carcinoma after curative tumor resection or ablation. Cancer Imaging 2019;19:11. 30813956 10.1186/s40644-019-0197-5 PMC6391838

[6] Zheng B-H Liu L-Z Zhang Z-Z. Radiomics score: a potential prognostic imaging feature for postoperative survival of solitary HCC patients. BMC Cancer 2018;18:1148. 30463529 10.1186/s12885-018-5024-z PMC6249916

[7] Cozzi L Dinapoli N Fogliata A. Radiomics based analysis to predict local control and survival in hepatocellular carcinoma patients treated with volumetric modulated arc therapy. BMC Cancer 2017;17:829. 29207975 10.1186/s12885-017-3847-7 PMC5718116

[8] Zheng J Chakraborty J Chapman WC. Preoperative prediction of microvascular invasion in hepatocellular carcinoma using quantitative image analysis. J Am Coll Surg 2017;225: 778-788.e1. 10.1016/j.jamcollsurg.2017.09.003 PMC5705269 28941728

[9] Cambridge University Press, Franklin J. Mapping Species Distributions: Spatial Inference and Prediction. 2010.

[10] Massada AB Syphard AD Stewart SI. Wildfire ignition-distribution modelling: a comparative study in the Huron–Manistee National Forest, Michigan. Int J Wildland Fire 2013;22:174–83.

[11] Greiner M Pfeiffer D Smith RD. Principles and practical application of the receiver-operating characteristic analysis for diagnostic tests. Prev Vet Med 2000;45:23–41. 10802332 10.1016/s0167-5877(00)00115-x

[12] Musio D De Francesco I Galdieri A. Diffusion-weighted magnetic resonance imaging in painful bone metastases: using quantitative apparent diffusion coefficient as an indicator of effectiveness of single fraction vs multiple fraction radiotherapy. Eur J Radiol 2018;98:01–6. 10.1016/j.ejrad.2017.10.025 29279145

[13] Liu X Li Y Qian Z. A radiomic signature as a noninvasive predictor of progression-free survival in patients with lower-grade gliomas. Neuroimage Clin 2018;20:1070–7. 30366279 10.1016/j.nicl.2018.10.014 PMC6202688

[14] Liu Z Zhang X-Y Shi Y-J. Radiomics analysis for evaluation of pathological complete response to neoadjuvant chemoradiotherapy in locally advanced rectal cancer. Clin Cancer Res 2017;23:7253–62. 28939744 10.1158/1078-0432.CCR-17-1038

[15] Cui Y Yang X Shi Z. Radiomics analysis of multiparametric MRI for prediction of pathological complete response to neoadjuvant chemoradiotherapy in locally advanced rectal cancer. Eur Radiol 2019;29:1211–20. 30128616 10.1007/s00330-018-5683-9

[16] Wu J Liu A Cui J. Radiomics-based classification of hepatocellular carcinoma and hepatic haemangioma on precontrast magnetic resonance images. BMC Med Imaging 2019;19:23. 30866850 10.1186/s12880-019-0321-9 PMC6417028

[17] Li Z Mao Y Li H. Differentiating brain metastases from different pathological types of lung cancers using texture analysis of T1 postcontrast MR. Magn Reson Med 2016;76:1410–9. 26621795 10.1002/mrm.26029

[18] Wang Q Zhou S Court LE. Radiomics predicts clinical outcome in primary gastroesophageal junction adenocarcinoma treated by chemo/radiotherapy and surgery. Phys Imaging Radiat Oncol 2017;3:37–42.

[19] Ding J Xing Z Jiang Z. CT-based radiomic model predicts high grade of clear cell renal cell carcinoma. Eur J Radiol 2018;103:51–6. 29803385 10.1016/j.ejrad.2018.04.013

[20] Braman NM Etesami M Prasanna P. Intratumoral and peritumoral radiomics for the pretreatment prediction of pathological complete response to neoadjuvant chemotherapy based on breast DCE-MRI. Breast Cancer Res 2017;19:57. 28521821 10.1186/s13058-017-0846-1 PMC5437672

[21] Buizza G Toma-Dasu I Lazzeroni M. Early tumor response prediction for lung cancer patients using novel longitudinal pattern features from sequential PET/CT image scans. Phys Med 2018;54:21–9. 30337006 10.1016/j.ejmp.2018.09.003

[22] Jiang Y Yuan Q Lv W. Radiomic signature of 18F fluorodeoxyglucose PET/CT for prediction of gastric cancer survival and chemotherapeutic benefits. Theranostics 2018;8:5915–28. 30613271 10.7150/thno.28018 PMC6299427

[23] Yang F Young LA Johnson PB. Quantitative radiomics: validating image textural features for oncological PET in lung cancer. Radiother Oncol 2018;129:209–17. 30279049 10.1016/j.radonc.2018.09.009

[24] Sala E Mema E Himoto Y. Unravelling tumor heterogeneity using next-generation imaging: radiomics, radiogenomics, and habitat imaging. Clin Radiol 2017;72:03–10. 10.1016/j.crad.2016.09.013 PMC5503113 27742105

[25] Larue RT Van De Voorde L van Timmeren JE. 4DCT imaging to assess radiomics feature stability: an investigation for thoracic cancers. Radiother Oncol 2017;125:147–53. 28797700 10.1016/j.radonc.2017.07.023

[26] Guo W Li H Zhu Y. Prediction of clinical phenotypes in invasive breast carcinomas from the integration of radiomics and genomics data. J Med Imaging 2015;2:041007. 10.1117/1.JMI.2.4.041007 PMC4718467 26835491

[27] Yang HX Feng W Wei JC. Support vector machine-based nomogram predicts postoperative distant metastasis for patients with oesophageal squamous cell carcinoma. Br J Cancer 2013;109:1109–16. 23942069 10.1038/bjc.2013.379 PMC3778272

[28] Vallières M Freeman CR Skamene SR. A radiomics model from joint FDG-PET and MRI texture features for the prediction of lung metastases in soft-tissue sarcomas of the extremities. Phys Med Biol 2015;60:5471–96. 26119045 10.1088/0031-9155/60/14/5471

[29] Parmar C Grossmann P Bussink J. Machine learning methods for quantitative radiomic biomarkers. Sci Rep 2015;5:13087. 26278466 10.1038/srep13087 PMC4538374

